# The Standard of Medical Education in America

**Published:** 1874-05

**Authors:** 


### Editorial.

### The Standard of Medical Education in the United States.

Our valuable cotemporary the *Philadelphia Medical Times* has raised its voice against the low standard of Medical Education in America and the ease with which John Smith, "Cooper," can be converted into John Smith "M. D. Physician and Surgeon." In a recent number it says: "If we omit Brown-Sequard—a cosmopolitan bird of passage—Leipsic, a town about the size of Providence, contributes to the world in a single year more in physiology that is worth the looking at than does America in a decade." We admit much of truth in this, but fear that the Editor has overlooked the works of Flint and Dalton, and the many valuable physiological facts which have emenated from this country in the past few years.

We know that a young man going from the work-bench, the plow or the anvil, with but the rudiments of the English language picked up in two or three winters "schooling" can, if he shows a certificate of having *read* medicine three years and attended two courses of lectures, by passing a reasonably easy examination, get a diploma, placing him on a par as far as the public is concerned with the student who has spent years in German Universities and French Hospitals. How is it to be remedied?

This is a question which requires much consideration and may be answered in several ways. Let us suggest one cause and it may point to a remedy.

America is known as a fast country. Our people are always in a hurry; and never go slow when it *pays* better to go fast. This applies to the Medical student as well as to the Broker on Wall Street. A young man enters the profession, let us say, with a fine preliminary education. He reads medicine under the direction of some physician during the summer and in the winter attends some good Medical College. He graduates at the end of three years with an average knowledge of Medical Science as far as it is included in the text books in general use. Having been admitted into the high calling of his profession his ambition leads him on, he determines to investigate, to experiment and discover, if possible, new facts in Medical Science. He hears the name of Brown-Sequard quoted as authority and is desirous of emulating him. His fellow members of the profession look, some perhaps with admiration but in silence, more with pity and treat him with laughter and ridicule that he should spend his time so foolishly. He soon finds out his mistake. A little experience in waiting for practice in his profession and fame as a scientific investigator satisfies him that his labors are not appreciated. He sees his classmate, who slept on the back seat during lectures, and who only got his diploma by dint of lucky guessing, growing daily into a paying practice. He observes that those who at one moment are consulting Brown-Sequard and other celebrities, the next call in the services of some charm doctor or other itinerant quack. He finds the rooms of an ignorant "female doctor" who cannot tell the first fact in anatomy or physiology crowded by ladies of wealth and culture, and finds the newspapers full of laudations of Dr. so and so who cures all diseases as if by magic.

Turning from his observations in sorrow in disgust, he lays aside his scalpel and microscope and falls into the well worn ruts of every day practice. It is not the action of the lower orders of society, who might be expected to see no difference between the "M. D." appended to his name and that affixed to his half-educated fellow, which blights his hope of succeeding in practice as a physician of eminent scientific attainment. It is rather the higher and more educated classes which are to blame, men of authority in church and state who foster and encourage these ignorant charlatans.

When he sees men and women to whom he had given credit for intelligence and culture, "fairly worshiping" (we quote from the l ps of one who is a devotee) an ignorant country farmer, who can not tell the first fact concerning any department of medicine, and believing that he, through the spiritual agency of a dead Indian, exerts a controlling influence over their diseases, even when miles away, is there any encouragement for him in perfecting his medical attainments or in spending the best years of his life in severe unrequiting study. He says No! Until the American people are educated to worship elsewhere than at the shrine of an Indian Clairvoyant, until they can recognize true merit and are bold enough to cast aside ignorant pretenders there will be no encouragement to any but the few favored by fortune or circumstance to make scientific m dicine their pursuit.

It is an aphorism in Civil Economy that the supply equals the demand; when the public demand physicians of scientific culture the supply will be met by American physicians and schools, until they do demand such, the standard of Medical Education must remain where it is.

